# Letters to the Editor

**Published:** 1987-11

**Authors:** Edward C. Hamlyn

**Affiliations:** Rutt House Ivybridge Devon PL21 0DQ


### Letters to the Editor

### Pollution and Allergy.

Sir,
Dr. David's compulsion to defend our profession's shortcomings in the field of allergy is perhaps preventing his attention from focusing on a major problem now facing the whole of medical practice.

We are leaving an era in which germs dominated the medical scene and have entered an age of pollution. Pollution can damage the immune system and impair the body's defence against pollution. Once the immune system is damaged the person starts to develop allergies.

Allergy as an aetiological factor in the mechanism of illness is now so widespread as to justify the screening of all patients for allergy as an initial step in modern diagnosis.

Dr. David refers to pollution as the cause of today's runaway epidemic of allergic disorders. He might be correct. We cannot ignore the increase in environmental pollution and it may be no coincidence that allergic illness increases in step with pollution.

A possible explanation for the medical profession dragging its feet in the task of assessing the importance of pollution in the current medical scene is the fact that the highest levels of pollution entering the human body are administered by doctors. In terms of tonnage the greatest quantity of pollutants entering our bodies comes across the chemist counter on or off prescription.

Two wrongs do not make a right and we are very misguided in imagining we can undo the harm we do with drugs by using more drugs to treat illness caused by drugs. With rising 20% of hospital admissions diagnosed as iatrogenic illness it is time for a reappraisal of where we are going as a profession.

Dr. David does not give a direct answer to the question as to why doctors refuse to listen to what patients tell them about allergic illness, but when he reveals that he regards the psychiatrist as his guru when it comes to the field of allergy, I think he gives us his answer.

Patients today have come to hate and fear the psychiatrist. Psychiatry is unscientific, a hang over from the dark ages and the modern well read patient knows this only too well.

It was Dr. Richard Mackarness, himself a psychiatrist, who first discovered that our mental hospitals could be filled with undiagnosed cases of masked allergy.

The implications of what Mackarness wrote in his books is such bad news for psychiatry that he is now out in the cold.

Sooner or later Dr. David and the whole medical profession will be called upon to face up to these issues. Let us do it in our own time at our own instigation instead of waiting until events overtake us.

Yours sincerely,

Edward C. Hamlyn, MB ChB.

Rutt House

Ivybridge

Devon PL21 0DQ

